# Intra-articular Injection of Baicalein Inhibits Cartilage Catabolism and NLRP3 Inflammasome Signaling in a Posttraumatic OA Model

**DOI:** 10.1155/2021/6116890

**Published:** 2021-09-02

**Authors:** Hui Bai, Rui Yuan, Zhiheng Zhang, Lin Liu, Xinyu Wang, Xiaopeng Song, Tianwen Ma, Jilang Tang, Chunpeng Liu, Li Gao

**Affiliations:** ^1^College of Veterinary Medicine, Northeast Agricultural University, Heilongjiang 150030, China; ^2^College of Animal Science and Technology, Zhongkai University of Agriculture and Engineering, Guangzhou 510225, China

## Abstract

Baicalein has been shown to have chondroprotective potential in vitro. However, its effect on disease modification in osteoarthritis (OA) is largely unknown. The present study is aimed at determining whether baicalein could slow the progression of OA and inhibit OA-related inflammation in a rat model of destabilization of the medial meniscus (DMM) and the underlying mechanisms. The rats subjected to DMM surgery were treated with baicalein (0.8, 1.6, and 3.2 *μ*g/L, 50 *μ*L, once a week) by intra-articular injection for 6 weeks. Dexamethasone (0.4 mg/mL, 50 *μ*L, once a week) was used as a positive control. Histologic grading of cartilage degeneration was performed using the Osteoarthritis Research Society International (OARSI) recommended grading system (on a scale of 0-6). The expression levels of molecules associated with cartilage homeostasis and inflammatory cytokines were analyzed; moreover, the NLRP3 inflammasome activation and cartilage oxidative stress-associated molecules were determined. Baicalein treatment reduced the OARSI score and slowed OA disease progression in a dose-dependent manner within a certain range. Compared with DMM rats, intra-articular injection of baicalein led to (1) reduced levels of inflammatory mediates such as IL-1*β* and TNF-*α*, (2) reduced immunochemical staining of MMP-13 and ADAMTS-5, (3) suppressed immunochemical staining loss of type II collagen, (4) reduced expression of cartilage degradation markers including CTX-II and COMP in urine, and (5) inhibited NLRP3 inflammasome activation rather than regulated expression of SOD, GSH, and MDA. In contrast to the administration of baicalein, dexamethasone injection showed similar effects to slow OA progression, while dexamethasone inhibited NLRP3 inflammasome partly through decreasing levels of SOD, GSH, and MDA. This study indicated that baicalein may have the potential for OA prevention and exerts anti-inflammatory effects partly via suppressing NLRP3 inflammasome activation without affecting oxidative stress-associated molecules, and inhibition of cartilage catabolism enzymes in an OA rat model.

## 1. Introduction

Osteoarthritis (OA) is the most common arthritis and degenerative disease of the articular joints involving the articular cartilage, subchondral bone, and synovium and characterized by joint dysfunction, chronic pain, and disability [1]. The only current therapeutic option for OA is pain-relief drugs, such as paracetamol and nonsteroidal anti-inflammatory drugs (NSAIDs) [2]. However, safety is focused due to gastrointestinal injury and cardiovascular events for long-term administration of NSAIDs [3].

In recent years, a variety of plant extracts, such as curcumin [4], L-theanine [5], and resveratrol [6], have been found to exert therapeutic or preventive effects on OA progression. Baicalein, a major flavonoid constituent in the plants of genus Scutellaria (Lamiaceae) [7], is found in abundant quantity in the root of S. baicalensis. Baicalein have been found to exhibit several pharmacological activities, such as antioxidant [8], anticancer [9], anticardiovascular [10], antidiabetic [11], and anti-inflammatory [12] activities. Evidence from several studies suggested that baicalein is effective in mitigating OA chondrocyte apoptosis and inflammation in vitro. For example, baicalein has been shown to alleviate the inflammatory process by reducing the expression of inflammatory cytokines such as IL-6, IL-8, and iNOS, in contrast increasing IL-10 production [13] and inhibiting degrading enzymes of the extracellular matrix such as matrix metalloproteinase-3 (MMP-3) and MMP-13 in human chondrocytes and in IL-1*β*- and TNF-*α*-treated mouse articular cartilage explants [14]. Moreover, our recent in vitro study determined the effects of baicalein on IL-1*β*-induced primary chondrocytes of rats. The results showed that baicalein downregulated the elevated caspase-3 levels but promoted the expression of Bcl-2. Baicalein treatment significantly inhibited NF-*κ*B signaling pathway activation and suppressed apoptosis and matrix degradation, indicating that baicalein might exert OA protective effect [15].

As is known, low-grade inflammation is believed to be involved in the development and progression of OA [16]; moreover, inflammatory cytokines such as IL-1*β*, IL-18, and TNF-*α* have demonstrated overexpression in posttraumatic osteoarthritis (PTOA) patients [17] and animal models [1]. A number of studies demonstrated that elevated levels of IL-1*β* and TNF-*α* suppress the synthesis of type II collagen and aggrecan, furthermore stimulating chondrocytes to release MMP-1, MMP-3, MMP-13, and disintegrin metalloproteinase with thrombospondin motifs (ADAMTS), which are major chondrocyte catabolic components to degrading the cartilage extracellular matrix [18–21]. Interestingly, it was determined that activation of inflammasome plays a key role in proinflammatory cytokine production, especially IL-1*β*. As a most studied inflammasome, NLRP3 inflammasome (NLR family, pyrin domain containing 3; NLR refers to “nucleotide-binding domain, leucine-rich repeat”) is responsible for the activation of caspase-1 and caspase-5, which leads to the secretion of IL-1*β* and IL-18, in turn causing pyroptosis [22]. Much higher levels of NLRP3 are detected in the OA synovium than in normal, subsequent activated caspase-1, and the release of IL-1*β* and IL-18 by pyroptotic chondrocytes triggers the inflammatory cascade, leading to cartilage degradation and chondrocyte death [23, 24]. There are various NLRP3 activators, such as mitochondrial damage, lysosomal damage, cytosolic K+ efflux, and reactive oxygen species- (ROS-) induced cell oxidative stress [25]. Several lines of evidences suggest that oxidative stress, which contributes to the imbalance of the expression of antioxidant enzymes and ROS scavenging systems, causes abnormalities in cartilage and bone metabolism, aggravating cartilage degradation [26]. Whether baicalein exerts its anti-inflammatory effects through NLRP3 inflammasome and the role of oxidative stress in the activation of NLRP3 during baicalein treatment are still unknown.

In a word, the chondrocyte protective effects and the underlying mechanism of baicalein for OA have not been well investigated. In our study, dexamethasone was used as a positive control due to its exact effect on OA disease modification through intra-articular injection [27], and the anti-inflammatory properties of dexamethasone have been considered [28]. Based on the above properties of baicalein, we aim to study the efficacy of baicalein compared to dexamethasone and the possible mechanism using a DMM-induced rat OA model in vivo.

## 2. Materials and Methods

### 2.1. Experimental Design

Rats were housed on a standardized pelleted diet and supplied with tap water. All animal experiments were carried out in accordance with the guidelines of the China Ethical Committee for Animal Experiments. OA was induced in 8-week-old male SD rats (bought from the laboratory animal center of Liaoning Changsheng Biotechnology Co., Ltd., China) by DMM surgery of the right knees as described [29]. Sham surgery was performed on the right knee joint using a separated group of rats as the control. Rats were randomly divided into 6 groups (*n* = 8/group): (1) DMM group, (2) sham group (vehicle control), (3) baicalein (0.8 *μ*g/L, 50 *μ*L), (4) baicalein (1.6 *μ*g/L, 50 *μ*L), (5) baicalein (3.2 *μ*g/L, 50 *μ*L), and (6) dexamethasone (DXM, 0.4 mg/mL, 50 *μ*L). After 1 week, rats in the treatment group received baicalein or dexamethasone (once a week) postoperatively via articular cavity injection for 6 weeks. Baicalein (Sigma-Aldrich, St. Louis, MO, USA) was dissolved in DMSO, and further dilutions were prepared in saline. In the control group, rats were injected with 2% DMSO (in saline) only. The dose of baicalein is determined based on our in vitro experiments and preliminary experiments [15]. Three different doses are set to avoid a single dose that is not effective, so as to choose a dose that can work.

After the last treatment, euthanasia was performed on the rats, and serum and urine samples were collected for the ELISA assay. Three out of 8 rats were chosen randomly of each group to observe the gross morphology, and subsequently, the articular cartilage was collected to detect the levels of MMP-13 and ADAMTS-5, and the experiment was repeated three times. Another 5 rats were used for pathological analysis, and the complete knee joints were immobilized in 4% paraformaldehyde.

### 2.2. Histological Examination

The knee joint in each group (*n* = 5/group) was placed in a bucket which was filled up with decalcification solution (Servicebio Biotechnology Co., Ltd, Wuhan, China, G1105) and then put in a constant temperature shaker. The replacement period of the decalcification solution is 2-3 d. The degree of decalcification is observed every two days. If the needle can be moved, the tissue is cut with a Lycra blade according to the material requirements, which can accelerate the softening speed. The dehydrating process was performed in gradient alcohol, and the intact flexed joint was embedded in paraffin. Then, serial sagittal sections were obtained across the entire knee joint. Slides were stained for sulfated glycosaminoglycans (GAGs) with Safranin O/Fast Green. Histologic grading of cartilage degeneration was performed using the Osteoarthritis Research Society International (OARSI) recommended grading system (on a scale of 0-6) [30], and histological analyses were performed by two experienced observers and double-blinded.

### 2.3. Immunohistochemical Assessment

After 6-week intra-articular injection, the complete right knee of every group (*n* = 3/group) was collected for the histologic section, and the procedures were in accordance with the histological examination. After deparaffinization, the sections were rinsed with 0.3% H_2_O_2_ in 60% methanol for 30 min and permeabilized with 0.1% Triton X-100 in phosphate-buffered saline (PBS) for 20 min. The section then was incubated in goat serum in PBS for nonspecific adsorption. For immunohistochemistry, sections were incubated with (1) anti-rabbit polyclonal antibody directed at collagen II (1 : 400; Abcam, ab34712), (2) anti-MMP-13 mouse monoclonal antibody (1 : 150; Novus, OTI2D8), (3) anti-ADAMTS-5 rabbit polyclonal antibody (1 : 100; Novus, NBP2-15286), (4) anti-NLRP3 rabbit polyclonal antibody (1 : 200; ABclonal, A12694), or anti-caspase-1 rabbit polyclonal antibody (1 : 150; ABclonal, A0964). Horseradish peroxidase- (HRP-) conjugated secondary antibody was applied and stained with a diaminobenzidine (DAB) kit. The positive stained chondrocytes in three central regions of articular cartilage were counted using Image-Pro Plus version 6.0 software.

### 2.4. Measurements of Inflammatory Cytokines

Serum samples from eight rats were collected, and concentrations of IL-1*β* and TNF-*α* were measured using specific rat enzyme-linked immunosorbent assay (ELISA) kits (Huijia Biotechnology Co., Ltd., Nanjing, China) according to the manufacturer's instructions.

### 2.5. Measurements of Cartilage Catabolism Protein

The cartilage of knee joints (*n* = 3/group) was collected, and the cartilage was ground with PMSF : RIPI (1 : 99) (Beyotime, China) under low temperature for 30 min and then centrifuged at 12000 rpm. 200 *μ*L of lysis buffer was added per 100 mg cartilage sample. The supernatant was collected for measuring the levels of MMP-13 and ADAMTS-5 via the ELISA kit (Huijia Biotechnology Co., Ltd (China). Rat urine samples (*n* = 8/group) were collected and centrifuged at 10,000 × g for 15 min within 1 h. The supernatant was stored at −80°C until future analysis. The concentrations of COMP and CTX-II (Jingmei Co., Ltd., Jiangsu, China) were measured in urine according to the manufacturer's instructions.

### 2.6. Measurements of SOD, GSH, and MDA Levels in Serum

Serum levels of superoxide dismutase (SOD), glutathione peroxidase (GSH), and malonaldehyde (MDA) were examined using detection kits (Beyotime Biotechnology Co., Ltd., Shanghai, China) according to the manufacturer's instructions.

### 2.7. Statistical Analysis

All statistical analyses were performed using SPSS 22.0 software, and the results are expressed as the mean ± standard deviation (SD). One-way analysis of variance (ANOVA) was used for comparisons between groups, and *p* < 0.05 was considered statistically significant. Multiple comparisons between groups were performed using a post hoc Tukey test. Histological analyses were performed by two experienced investigators who were double-blinded.

## 3. Results

### 3.1. Changes in Gross Morphology of the Joints in the DMM-Induced OA Model

**Figure 1:**
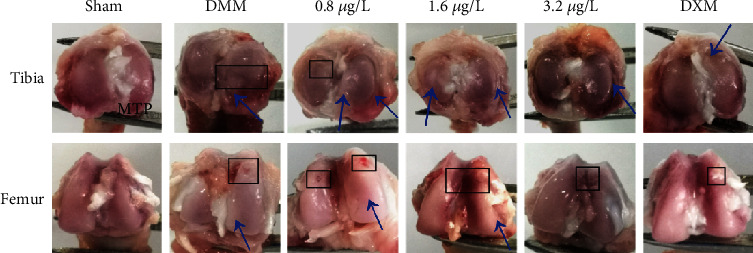
Comparisons of gross appearance of femoral condyle and tibial plateau in rat DMM osteoarthritis models and different doses of baicalein- or DXM-treated rats at 7 weeks postsurgery. The condyle of the distal femur showed a rough joint surface at the intercondylar space and osteochondral defect (black box). The medial tibial plateau (MTP) exhibited osteophyte formation (blue arrows) (*n* = 3).

A healthy articular surface was observed in both femur and tibia in sham cartilage (Figure 1). There were obvious macroscopic changes in the appearance of the right joints in the OA model at 7 weeks postsurgery. DMM cartilage was abraded and contained fibrotic tissue, and the cartilage showed significant osteophyte formation at the medial tibia plateau (MTP) and the intercondylar space of the femur (Figure 1). Moreover, the cartilage of baicalein-treated rats with 0.8 and 1.6 *μ*g/L exhibited no detectable macroscopic difference compared to DMM cartilage. In contrast, the joints administrated with 3.2 *μ*g/L baicalein or dexamethasone showed significantly milder injuries than DMM joints, including cartilage opacity and roughness (Figure 1).

### 3.2. Baicalein Ameliorates OA Progression in DMM-Induced Rat Model

**Figure 2:**
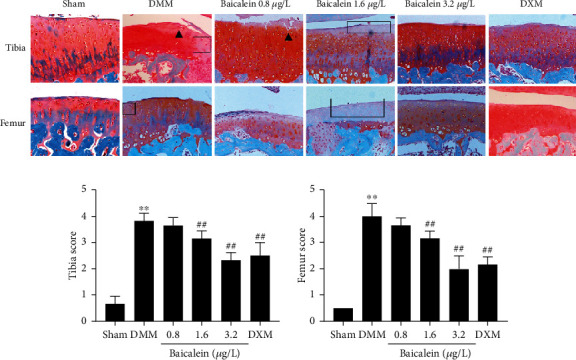
Baicalein treatment ameliorated OA cartilage lesions. (a) Representative images of cartilage in the tibial plateau and femur (right knee of rats). Safranin O-Fast Green staining was performed to evaluate the relative content of proteoglycan (red color in Safranin O staining) and articular cartilage degeneration. Cartilage erosion and cartilage fibrillation were shown as black arrows. Proteoglycan loss was exhibited as a black box. Original magnification ×100 (*n* = 5 for each group). (b) OARSI scores assessed by two experienced observers (*n* = 5 for each group). Values are the mean ± SD; ^∗∗^*p* < 0.01 vs. sham group. ^##^*p* < 0.01 vs. DMM group.

We determined the efficacy of baicalein on DMM-induced OA by evaluating the structural integrity of the articular cartilage using microscopy following Safranin O staining and OARSI score for the whole joint. Sham joints showed no obvious surface changes and matrix disturbance. Rats subjected to DMM displayed surface discontinuity, cartilage erosion, cartilage fibrillation at the midzone of cartilage, and matrix degradation (loss of Safranin O staining) (Figure 2(a)). The OARSI scores in DMM cartilage of both tibial plateau and femur were significantly increased compared to sham joints. In contrast, less Safranin O loss and cartilage fibrillation were observed in both baicalein (3.2 *μ*g/L)-treated rats and DXM-treated rats (Figure 2(a)) with a significantly lower OARSI score compared to that in the vehicle-treated DMM group. Of note, although baicalein-treated rats with 0.8 *μ*g/L and 1.6 *μ*g/L also showed a decreased OARSI score as compared with vehicle-treated DMM rats (Figure 2(b)), the cartilage still showed loss of Safranin O staining and cartilage degeneration, including surface fibrillation, cartilage cyst formation, and disordered arrangement of cells (Figure 2(a)). As a positive control, DXM-treated rats with DMM also displayed less cartilage injury as compared with vehicle-treated DMM rats. Moreover, a similar OARSI score was observed in the DXM group to baicalein (3.2 *μ*g/L)-treated rats (Figure 2(b)).

### 3.3. Baicalein-Induced Improvement in Cartilage Metabolism

**Figure 3:**
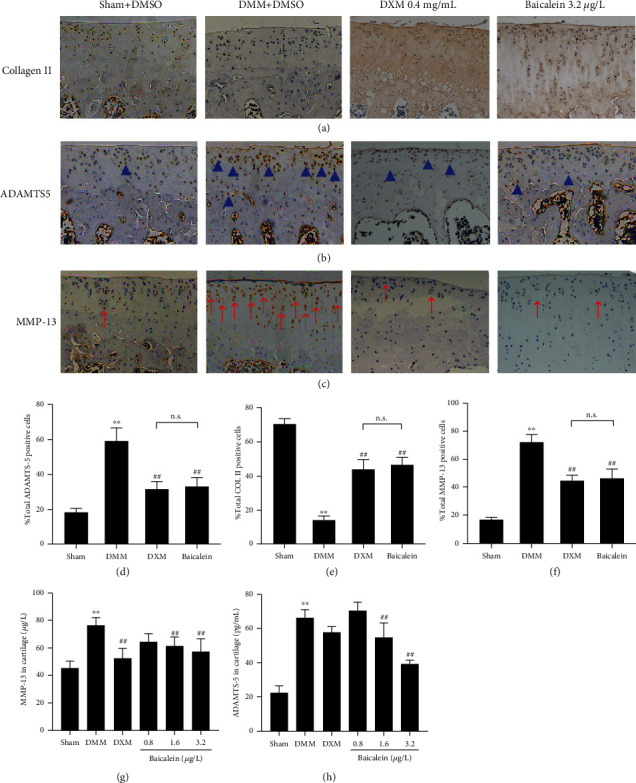
Effects of baicalein on cartilage Col II levels and degradation enzymes. (a–c) IHC analysis of Col II, ADAMTS-5, and MMP-13, respectively, in the tibial plateau of right knee joints (*n* = 3 for each group). Positive cells of ADAMTS-5 were shown as blue arrows (b), and MMP-13 positive cells were shown as red arrows (c). Original magnification ×100. (d–f) Quantitative data of the percentage of ADAMTS-5, Col II, and MMP-13 positive cells (*n* = 3 for each group). (g, h) ELISA assay of MMP-13 and ADAMTS-5 in cartilage tissues (*n* = 3 for each group). Values are the mean ± SD; ^∗∗^*p* < 0.01 vs. sham group. ^##^*p* < 0.01 vs. DMM group. n.s.: no significance.

To explore whether baicalein affects cartilage metabolism, we performed immunohistochemistry (IHC) analyses to examine the expression of Col II, ADAMTS-5, and MMP-13 and ELISA to measure the expression of ADAMTS-5 and MMP-13 in cartilage tissues. The results showed that the expression of Col II was markedly lower in the DMM group as compared with the sham control. After baicalein (3.2 *μ*g/L) and DXM treatment, the ratio of cells with positive Col II in the cartilage was significantly increased (Figures 3(a) and 3(d)). In contrast, after DMM surgery, the expression of ADAMTS-5 and MMP-13 in cartilage by IHC and ELISA was significantly increased. However, baicalein (3.2 *μ*g/L) largely decreased the expression of ADAMTS-5 and MMP-13 as compared with vehicle-treated DMM rats (Figures 3(b), 3(c), 3(e), and 3(f)). Furthermore, in baicalein-treated rats (both 1.6 *μ*g/L and 3.2 *μ*g/L), the levels of ADAMTS-5 (*p* = 0.004 and *p* < 0.001) and MMP-13 (*p* = 0.009 and *p* < 0.001) (detected by the ELISA assay) in cartilage tissue were also significantly decreased as compared with vehicle-treated DMM rats (Figures 3(g) and 3(h)). In addition, administration of DXM also reduced the positive ratio of MMP-13 and ADAMTS-5 in articular cartilage and exerted similar effect with baicalein (3.2 *μ*g/L) in IHC results. Furthermore, the ELISA results showed that the levels of MMP-13 and ADAMTS-5 were decreased by baicalein (both 1.6 *μ*g/L and 3.2 *μ*g/L) and DXM, even though the reduction effect of DXM on ADAMTS-5 was just slight.

### 3.4. Baicalein Treatment Downregulates the NLRP3 Inflammasome Pathway and TNF-*α* Levels

**Figure 4:**
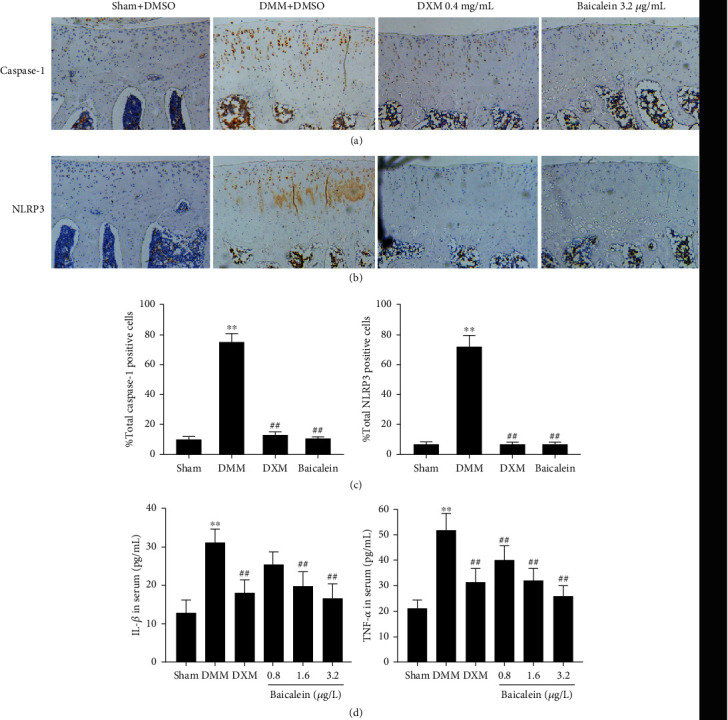
Effects of baicalein on NLRP3 inflammasome pathway and serum expression of inflammatory mediators. (a) IHC analysis of caspase-1 in the tibial plateau of the right knee joint (*n* = 3). (b) IHC analysis of NLRP3 in the tibial plateau of the right knee joint (*n* = 3). (c) Data analysis of IHC results. (d) ELISA analysis of the levels of IL-1*β* and TNF-*α* in serum (*n* = 8 for each group). Values are the mean ± SD; ^∗∗^*p* < 0.01 vs. sham group. ^##^*p* < 0.01 vs. DMM group.

To evaluate the activation of NLRP3 inflammasome in DMM-induced OA cartilage, the level of IL-1*β*, the effector of NLRP3 inflammasome, was found markedly increased (1.6 *μ*g/L, *p* < 0.001; 3.2 *μ*g/L, *p* < 0.001, respectively) in serum of the DMM rats (Figure 4). Notably, the expression of NLRP3 and caspase-1 was significantly higher in DMM cartilage. In contrast, the expression levels of NLRP3, caspase-1, and IL-1*β* were markedly decreased after being treated with 3.2 *μ*g/L baicalein or DXM (Figures 4(a)–4(d)).

In addition, we observed that the levels of TNF-*α* were also decreased (0.8 *μ*g/L, *p* < 0.004; 1.6 *μ*g/L, *p* < 0.001; and 3.2 *μ*g/L, *p* < 0.001, respectively) as compared with the DMM group (Figure 4(d)). Similarly, DXM (positive control) administration reduced the expression of TNF-*α* (*p* < 0.001). In a word, baicalein treatment inhibited the expression of NLRP3 and caspase-1 in cartilage and reduced the serum levels of IL-1*β* and TNF-*α*, and a higher dose of baicalein (3.2 *μ*g/L) exhibited comparable effects with DXM.

### 3.5. Baicalein Treatment Dose not Affect the Antioxidant System in OA

**Figure 5:**
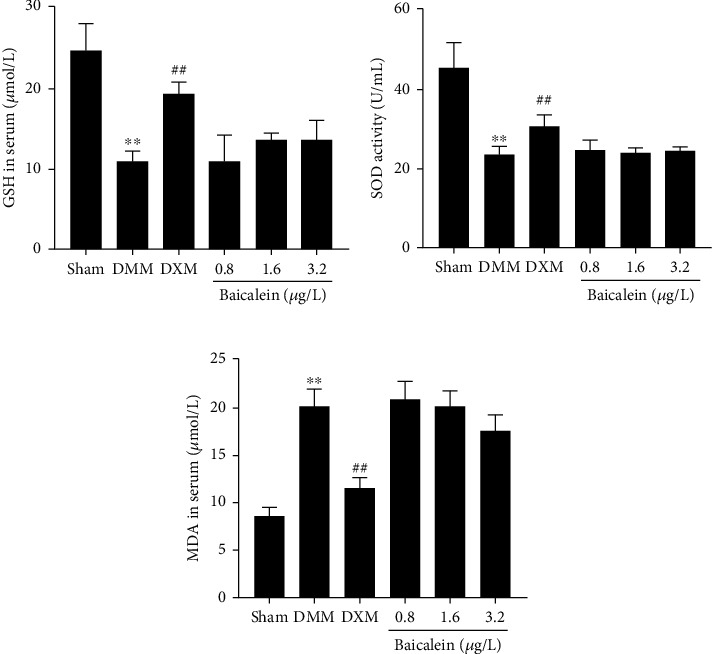
Effects of baicalein supplementation on serum levels of GSH, SOD, and MDA in rats with DMM-induced OA (*n* = 8 for each group). Values are the mean ± SD; ^∗∗^*p* < 0.01 vs. sham group. ^##^*p* < 0.01 vs. DMM group.

Changes in MDA levels (lipid peroxidation product) and antioxidant enzymes (SOD, GSH) are a sign of oxidative stress status. As oxidative stress is reported responsible for NLRP3 activation, we subsequently detected the changes of MDA, SOD, and GSH in serum. The reductions in the levels of GSH and SOD were observed in OA rats induced by DMM surgery (Figures 5(a) and 5(b)). In contrast, the levels of MDA were significantly increased in DMM rats (Figure 5(c)). Intra-articular injection of dexamethasone (DXM) highly increased the concentrations of GSH and SOD, instead of decreasing MDA in serum. Notably, six-week baicalein injection after DMM induction had no significant effect on serum levels of GSH, SOD, and MDA, which were tightly related to oxidative stress (Figures 5(a)–5(c)).

### 3.6. Baicalein Treatment Reduces Urine Levels of OA Biomarkers

**Figure 6:**
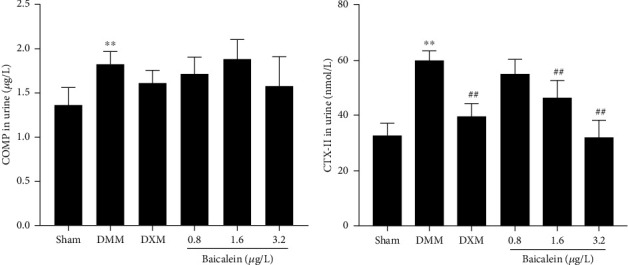
Effects of baicalein on the levels of urine biomarkers. (a, b) ELISA analysis of the levels of COMP and CTX-II in urine (*n* = 8 for each group). Values are the mean ± SD; ^∗∗^*p* < 0.01 vs. sham group. ^##^*p* < 0.01 vs. DMM group.

To investigate whether baicalein also shows the potential effect of degradation products of ECM, we examined the effect of intra-articular injection of baicalein on the production of COMP and CTX-II, which are key biomarkers of ECM hydrolysis. The results showed that the urine levels of COMP (*p* = 0.007) and CTX-II (*p* < 0.001) significantly increased in the DMM group as compared to sham-operated rats. However, no significant change of COMP was observed in both baicalein and DEX treatment groups (Figure 6(a)). Levels of CTX-II was significantly inhibited in the DEX group (*p* < 0.001) and baicalein group (1.6 *μ*g/L, *p* = 0.002 and 3.2 *μ*g/L, *p* < 0.001, respectively) as compared with the DMM-induced OA group (Figure 6(b)).

## 4. Discussion

Recently, most of plant extracts were applied to OA prevention, including L-theanine [5], sesamin [31], and curcumin [4] in animal OA models. OA disease-modifying drugs were urgently needed, while there are still few effective drugs available to alleviate the disease progression. A drug, such as baicalein, acting on the inhibition of chondrocyte apoptosis via NF-*κ*B signaling and reduction of inflammatory cascades in in vitro chondrocytes [13] and whether it has the potential to treat OA disease and the underlying mechanism were still not well studied. Our present study found that intra-articular injection of baicalein slowed down the progression of OA disease and ameliorated arthritic symptoms and cartilage erosion in a posttraumatic OA rat model.

Various animal models were demonstrated to be able to well simulate human OA progression and were widely used in a number of OA disease remission drug studies, for example, spontaneous models in guinea pigs, surgery-induced traumatic models, and intra-articular injection of drugs, such as iodine acetate, papain, and collagenase [32]. We established a DMM model, a sufficiently sensitive model to mimic human OA progression [29]. Moreover, the DMM-induced OA model was considered easier to operate than the anterior cruciate ligament (ACL) transection used in our previous study [5]. In the present study, we found deteriorated articular cartilage damage and severe joint inflammation in the DMM-induced OA model rats, which is consistent with previous studies [29], whereas our results in this study showed more severe histological changes accompanied by a 4.0 ± 0.86 OARSI score in DMM-induced cartilage 7 weeks postsurgery, when compared to the features described by Kung et al. in 2019 [33] and Ramos et al. in 2020 [34], while their results of cartilage histological scores were from 3.3 to 3.6. The reason of these differences might attribute to the technical challenge [35] and the number of joints being assessed; therefore, a large sample size was necessary in future studies.

Evidence suggested that the NLRP3 inflammasome is involved in OA development, which contributed to the degradation of articular cartilage and joint inflammation [36]. Vandanmagsar et al. observed that obesity-induced inflammation was prevented via NLRP3 ablation in mice, indicating the NLRP3 inflammasome may be related to OA progression [37]. Recent studies documented that the expression of NLRP3 was increased in patients with knee OA [23]. Baicalein has established potent anti-inflammatory effects via multiple mechanisms, for instance, reduction of NLRP3 inflammasome activation in LPS-induced macrophages [38]. In addition to macrophages, baicalein was shown to inhibit NLRP3 and inflammatory cytokines in N-methyl-4-phenyl-1,2,3,6-tetrahydropyridine- (MPTP-) stimulated microglia to induce PD-like pathology [39]. Consistently, our results showed that NLRP3 protein expression was significantly increased in the cartilage of OA rats compared to sham controls. Treatment with baicalein showed obvious effectiveness in inhibiting NLRP3 inflammasome activation.

Several studies have confirmed that assembly of the NLRP3 inflammasome cleaves procaspase-1 into an active form, leading to the maturation of proinflammatory cytokines, including IL-1*β* and IL-18 [40]. As well-documented inflammatory mediators, IL-1*β*, IL-18, and TNF-*α* have been proved to be involved in various disease progression, including OA [41]. Overproduction of IL-1*β* leads to autocrine production of degradation enzymes of ECM in early OA, such as MMP-3, MMP-13, and ADAMTS-4/5 through initiating transcription of NF-*κ*B signaling [42]. In our present study, rats subjected to DMM showed significantly increased expression of caspase-1 and IL-1*β*, while being suppressed by baicalein treatment. Moreover, our results of increased levels of MMP-13 and ADAMTS-5 and decreased levels of type II collagen in DMM-induced joints were consistent with the above study, suggesting the effects of IL-1*β* in promoting cartilage degradation. However, regarding the secretion of IL-1*β* by synovial cells or chondrocytes responsible for the pathogenesis of OA, the existing results were conflicting. Nasi et al. documented that IL-1*β*^−/−^ mice were not protected against cartilage erosion induced by DMM surgery [43]. On the contrary, diminished IL-1*β* by RNA interference-base reduction (siRNA) lowered the expression of OA-related cytokines such as IL-1*β*, TNF-*α*, and IL-8 [44]. The difference of these discrepant results may be due to the variability in the method of OA induction, difference of age, or sex of the animals.

NLRP3 activation is regulated by multiple signaling pathways, including potassium ion efflux, lysosomal destabilization, and ROS-induced mitochondrial dysfunction [45]. Multiple studies have confirmed that oxidative stress is an important risk factor for NLRP3 activation [46]. Abnormal ROS-induced oxidative stress signaling which has been documented in OA chondrocytes accompanied with articular injury [47] was involved in the activation of NLRP3 inflammasome [48]. A number of antioxidant enzymes including SOD and GSH were produced by chondrocytes to prevent an accumulation of ROS-mediated damage [49, 50]. SOD and GSH are known as two main antioxidants, which act as a ROS scavenger and are against MDA. MDA, a marker of lipid peroxidation, was highly expressed in arthritis animal models or OA patients [51]. To study a possible mechanism of whether baicalein decreased the NLRP3 protein level and downstream suppressed caspase-1 and IL-1*β* via inhibition of oxidative stress, we determined the expression of signs of oxidative stress status. The results in our study showed that the levels of antioxidative SOD and GSH were significantly decreased and those promoting oxidative MDA were increased in DMM-induced OA rats, while intra-articular injection of baicalein showed mild changes. The above results revealed that baicalein inhibited NLRP3 activation and exerted anti-inflammatory effects, which was independent of oxidative stress-related signaling pathways. Interestingly, we observed that the level of TNF-*α* in serum was increased after DMM surgery, while baicalein treatment led to a remarkable decrease. The previous study reported that TNF-*α* induced generation of ROS which was not able to activate NLRP3 inflammasome [52]. This suggests that a specific type of ROS is required or that ROS is not sufficient for NLRP3 inflammasome activation. The limitation of our study is that direct detection of ROS levels is necessary, and the specific inhibitors of NLRP3, caspase-1, or ROS signaling are needed in future research.

In our study, dexamethasone was selected as a positive control. Local administration of glucocorticoids (e.g., dexamethasone) has been used in the management of OA for a long time [53]. Intra-articular injection of dexamethasone has been reported to benefit lipopolysaccharide-induced equine synovitis [54]; furthermore, low-dose dexamethasone injection reduced the loss of proteoglycan and collagen, maintaining chondrocyte homeostasis [27]. In addition to powerful anti-inflammatory effects, dexamethasone has been reported to suppress the NF-*κ*B/NLRP3 pathway in ovalbumin-induced allergic asthma. In a mouse model of chronic obstructive pulmonary disease (COPD), dexamethasone not only downregulated NLRP3, caspase-1, and IL-1*β* but also upregulated the levels of SOD. In the present study, we found that intra-articular injections of dexamethasone significantly reduced NLRP3, caspase-1, and IL-1*β*. Interestingly, when compared with baicalein, dexamethasone treatment strongly suppressed the levels of MDA but upregulated SOD and GSH expression. Thus, the inhibition efficacy on NLRP3 inflammasome of dexamethasone would be greatly attributed to the regulation of oxidative stress in cartilage.

COMP is a pentameric glycoprotein of the thrombospondin family that is found abundantly in articular cartilage and also found in tendons and synovium [37]. It has been appreciated that COMP is important for the stabilization of extracellular matrix components, especially for the integrity of the collagen network [38]. Urinary CTX-II, a major downstream product of type II collagen breakdown, has been negatively correlated with the joint surface area and is an important predictor of joint damage. Our study showed the effects of baicalein and dexamethasone on the expression of COMP with no significant difference compared with DMM rats, while both of the two drugs suppressed the levels of CTX-II, MMP-13, and ADAMTS-5. The present results suggested that baicalein is available for OA treatment through inhibition of OA cartilage catabolism events almost identical to dexamethasone. The limitation of our study is that we assessed the effects of baicalein in a six-week period; the long-term effect of baicalein administration for OA treatment and the activity of baicalein via oral administration were necessary to examine. More specific roles of NLRP3 in baicalein-treated OA and the underlying mechanism of NLRP3 activation need further exploration.

## 5. Conclusions

Our data indicated that baicalein performed anti-inflammation effects and ameliorated cartilage injury in vivo. Baicalein exerts its efficacy at least partially by regulating the NLRP3-IL-1*β*-cartilage degradation axis, rather than inhibiting cartilage oxidative stress-associated molecules; meanwhile, similar suppression activity on inflammatory cytokines was observed when compared with dexamethasone. This is the first evidence that baicalein slowed OA progression in a rat OA model.

## Data Availability

All data generated or analyzed during this study are included in this published article.
